# Lack of Association between Variant rs7916697 in* ATOH7* and Primary Open Angle Glaucoma in a Saudi Cohort

**DOI:** 10.1155/2018/2148056

**Published:** 2018-11-01

**Authors:** Altaf A. Kondkar, Taif A. Azad, Faisal A. Almobarak, Ibrahim M. Bahabri, Hatem Kalantan, Khaled K. Abu-Amero, Saleh A. Al-Obeidan

**Affiliations:** ^1^Glaucoma Research Chair, Department of Ophthalmology, College of Medicine, King Saud University, Riyadh, Saudi Arabia; ^2^King Khaled University Hospital, King Saud University, Riyadh, Saudi Arabia

## Abstract

A case-control genetic association study was performed to investigate whether variant rs7916697 in* atonal bHLH transcription factor 7* (*ATOH7*), which has been previously reported to be associated with optic disc parameters and primary open angle glaucoma (POAG) in different ethnic groups, is a risk factor for POAG or any of its clinical phenotypes in a Saudi cohort. Genotyping of rs7916697 (G>A) variant was performed in 186 unrelated POAG cases and 171 unrelated nonglaucomatous controls of Saudi origin using real-time Taq-Man® assay. Genotypic and allelic association with POAG and its related clinical indices were evaluated. Demographic and systemic disease status did not differ significantly between POAG cases and controls. Association analysis between POAG cases and controls showed no significant genotype effect under additive (p=0.707), dominant (p=0.458), and recessive (p=0.554) models. Besides, the minor ‘A' allele frequency was 0.39 in POAG cases and 0.36 in controls with no significant distribution (p=0.406). In addition, there was no significant difference between genotypes and clinical phenotypes such as intraocular pressure and cup/disc ratio within the POAG group, or any age and sex adjusted genotype effect on the disease outcome in regression analysis. Variant rs7916697 in* ATOH7* is not associated with POAG or its clinical indices such as IOP and cup/disc ratio in a Saudi cohort.

## 1. Introduction

With an estimated heritability of 0.81 [1], primary open angle glaucoma (POAG) follows a complex multi-factorial inheritance pattern involving both genetics and environmental factors [2].* Atonal bHLH transcription factor 7* (*ATOH7*), located on cytogenic band 10q21.3-22.1, is a single exon gene that encodes transcription factor known to play a central role in differentiation of retinal ganglion cells (RGCs) and optic nerve formation [3]. Studies in animal model of glaucoma have demonstrated that abnormal ATOH7 expression results in an increased number of differentiated RGCs [4, 5]. Mice retinal progenitor cells have been demonstrated to express* Atoh7 *[6] and its regulation by* Pax6* in embryonic retina [7] is clinically relevant because* PAX6* gene mutations have been associated with various optic nerve abnormalities in humans [8]. Genome-wide association studies (GWAS) have previously reported strong association between optic disk parameters and variant rs7916697 near* ATOH7* in an Australian twin cohort [6], the Rotterdam study [9], and Singapore Asians [10] and very recently in Latino population [11]. A recent meta-analysis of GWAS within the International Glaucoma Genetics Consortium revealed that rs7916697, among others, significantly affected at least one of the optic disc parameters [12]. In addition, a suggestive protective association was also noted for rs7916697 in Afro-Caribbean Barbados population in POAG [13] indicating* ATOH7* as an important susceptibility gene associated with glaucoma and optic disc parameters. Despite strong evidence for involvement of* ATOH7* in pathogenesis of POAG the exact molecular mechanism(s) leading to glaucomatous damage of the optic nerve and the possibility of any interaction of* ATOH7* with other risk factors is still largely unknown.

Our previous study has shown that another polymorphism rs1900004 in* ATOH7*, also reported to be strongly associated with optic disk parameters and POAG [6, 9], was not associated with POAG in a small number of Saudi patients that we investigated [14]. The present study was performed in a different and almost double the number of sample cohorts of Saudi origin to investigate any association between variant rs7916697 in* ATOH7* and POAG or any of its clinical indices.

## 2. Material and Methods

### 2.1. Study Population

A case-control study was performed according to the principles of the Declaration of Helsinki for human research and all of the study participants provided a written informed consent. The study received ethical clearance from College of Medicine institutional review board committee at King Saud University (approval number # 08-657). Saudi patients with clinically confirmed diagnosis of POAG (n=186) and nonglaucomatous healthy controls (n=171) of the same ethnicity were recruited into the study at King Abdulaziz University Hospital, King Saud University, Riyadh, Saudi Arabia. The criteria for selection of patients and controls have been detailed elsewhere [15, 16]. A standardized ophthalmic examination was performed in all the participating patients that included measurement of intraocular pressure (IOP) by Goldmann applanation tonometry mounted at the slit lamp, examination of anterior chamber angles by gonioscopy, dilated pupil examination of the lens and fundus, and visual field testing by Humphrey automated field analyzer. POAG patients were diagnosed by glaucoma specialist ophthalmologist and satisfied the following diagnostic criteria: (1) presence of progressive glaucomatous damage at the optic disk or retinal nerve fiber layer changes, such as narrowing of the neuroretinal rim, diffuse thinning of retinal nerve fibre layer, or localized defects; (2) presence of visual field defects which are typical of glaucoma such as nasal step defect, arcuate or paracentral scotomata, or generalized tunnel vision; (3) bilaterally open anterior chamber angles as examined by gonioscopy; and (4) adult onset. Any secondary form of glaucoma cases such as pigmentary glaucoma, uveitic, pseudoexfoliation, and history of steroid use or ocular trauma was excluded. Ethically matched control subjects were selected from ophthalmology screening clinics and subjects with normal IOP (without anti-glaucoma medicine), open anterior chamber angles, and healthy optic disk on examination with no previous history of ocular disease(s) or ophthalmic surgeries. Patient or control subjects refusing to participate were excluded. Other details on history of systemic diseases, health awareness, and smoking habits were procured through medical records of the patients or personal interviews for controls.

### 2.2. Genotyping of rs7916697 in* ATOH7* Gene

DNA samples were obtained from peripheral EDTA blood using the illustra blood genomicPrep Mini Spin kit (GE Healthcare, Buckinghamshire, UK) and genotyped for rs7916697 G>A polymorphism near* ATOH7* gene (NG_031934.1) using the TaqMan® SNP Genotyping Assay (assay ID: C_27850155_10; Applied Biosystems Inc., Foster City, CA, USA) on ABI 7500 Real-Time PCR System (Applied Biosystems) using recommended cycling conditions as described previously [17]. Fluorescence was measured at annealing step and genotype calling was performed using the automated 2-color allele discrimination software on ABI 7500.

### 2.3. Statistical Analyses

SPSS version 22 (IBM Inc., Chicago, Illinois, USA) was used to perform the analyses. Chi-square analysis was used to test Hardy-Weinberg Equilibrium (HWE) and allelic/genotype association. Independent samples t-test, one-way ANOVA, and Kruskal-Wallis tests were used to detect the mean difference across genotypes/groups. Regression analysis was done to determine the effect of age, sex, and genotype on the disease (POAG) outcome. Odds ratios (OR) were calculated, confidence interval (CI) level was set to 95%, and a p value less than 0.05 was considered statistically significant.

## 3. Results

### 3.1. Demographic and Clinical Characteristics of the Participants

A total of 357 participants consisting of 186 POAG cases and 171 nonglaucomatous controls were included in this study. Except for family history of glaucoma (p=0.039), the mean age, gender distribution, smoking habits, and status of systemic diseases such as diabetes, hypertension, coronary artery disease, and hypercholesterolemia showed no significant difference between the two study groups (Table 1).

### 3.2. Genotype and Allelic Association with POAG

The genotypes did not deviate significantly from the HWE (p>0.05). Both the genotype and allele frequency of rs7916697 in* ATOH7* did not differ significantly between POAG cases and controls (Table 2). The minor ‘A' allele frequency was 0.39 in POAG cases and 0.36 in controls with no significant distribution between cases and controls (p=0.406). There was no significant genotype association with POAG as assessed by additive (p=0.707), dominant (p=0.458), and recessive models (p=0.554). In addition, there was no significant difference between genotypes and clinical phenotypes such as intraocular pressure and cup/disc ratio within the POAG group (Table 3), or any age and sex adjusted genotype effect on the disease outcome in regression analysis (Table 4).

## 4. Discussion

Variant rs7916697 is located in the 5′ untranslated region of* ATOH7*, an important candidate for human optic nerve aplasia and related clinical syndromes [3]. A genetic association between rs7916697 and risk of POAG in a Saudi cohort was investigated.

The minor allele frequency (MAF) of rs7916697'A' in* ATOH7* varies across different population ranging from 0.25 to 0.82 with an overall MAF of 0.44 (1000 Genomes Project, Ensembl database). The MAF in our Saudi cohort was 0.36 in controls and 0.39 in POAG cases. The observed MAF was higher than 0.27 reported in US Caucasians [18], similar to 0.38 reported in Latino study [11] and much lower than 0.76 observed in Afro-Caribbeans where ‘G' was reported to be the minor allele [13], highlighting an ethnic specific complex genetic etiology in POAG.

Genetic studies have reported both positive and negative association of variants in* ATOH7* and risk of POAG in different ethnic groups. Polymorphism rs7916697 in* ATOH7* was reported to be an important genetic determinant of optic disc size in a meta-analysis consisting of UK and Australian cohort (p=1.3x10^−10^) that explained 1.7% variation in the optic disc area in the UK cohort [6], implicating its pathophysiological role in POAG. Studies in Rotterdam and Singapore had identified significant association between rs7916697 and optic disc area (p=2x10^−15^) in Asians [10]. The Blue Mountains Eye Study and the Twins study also replicated a strong association at rs7916697 for optic disc size that was dependent on vertical cup/disc ratio (VCDR) [19]. Similarly, consistent with other findings, rs7916697 showed a borderline genome-wide significance (p=5.44x10^−8^) and was associated with decrease in VCDR in the Latino population [11]. In contrast, we did not find any significant association between rs7916697 and POAG or any of its clinical indices such as IOP, cup/disc ratio, and number of anti-glaucoma medications that serve as clinical markers of disease severity/progression. Consistent with our findings, rs7916697 was also not found to be significantly associated with POAG and other clinical indices in US Caucasians, where another variant rs1900004 in* ATOH7* was reported to influence optic disc area [18]. Similarly, a modest protective effect was reported for rs7916697 (allelic p* *=* *0.0096, genotypic p* *=* *0.01) for the ‘G' allele (OR* *=* *0.67; 95% CI* *=* *0.50−0.91) in Afro-Caribbean subjects. Though this finding failed to withstand correction for multiple testing, there was significant evidence for an interactive effect with rs1063192 (near* CDKN2B/AS1* on chromosome 9) [13].

The molecular mechanisms leading to the development and progression of optic nerve defect in POAG could be IOP- and/or non-IOP-dependent (RGC/optic nerve vulnerability-related). Genes affecting optic nerve quantitative traits such as optic disc area and VCDR may have a plausible role in pathogenesis of POAG by affecting developmental-related pathways [20].* ATOH7* gene encodes Math5, a protein that plays a central role in RGC differentiation [4] and thus may not be a major risk modulator or causal factor in IOP-related pathogenesis of late-onset POAG. A complex disease such as glaucoma can result from interactions between several genes. An additive effect of genetic variants associated with IOP, VCDR, and high or normal tension glaucoma [13, 21] cannot be ruled out in this study. In addition, we have previously shown that polymorphism rs1900004 in* ATOH7* was also not associated with POAG [14], indicating that specific genetic variants may be more enriched in one ethnic population than another, highlighting racial differences, and that* ATOH7* may not have a major role in POAG development/progression in this population. Nonetheless, the findings of our study require cautious interpretation. Considering the global MAF (from 1000 Genomes database) or the MAF observed in our Saudi cohort, the current sample size of the study has an estimated power of >80% (with alpha risk of 5%) to detect an odds ratio of 2.0. However, it will certainly require a much larger sample size to detect a 1.5-fold relative risk. As with other complex diseases, large sample sizes are required to ensure sufficient power to fully define the underlying genetic causal effect which may be a major limitation in this study. Besides, the role of other variants and gene-gene interaction also cannot be ruled out.

## 5. Conclusion

In an attempt to link this polymorphism with POAG among the Saudi cohort our study shows that variant rs7916697 in the* ATOH7* gene lacks significant association with POAG or related phenotypes such as IOP and cup/disc ratio. This observation needs further validation in a much larger sample population of clinically well-defined POAG patients potentially with age, gender, and ethnicity matched controls to assess the risk it may contribute to the development or progression of the disease in this population.

## Figures and Tables

**Table 1:** Demographic and clinical characteristics of POAG cases and controls genotyped for SNP rs7916697.

**Variables**	**Controls** **(n = 171)** **No. (**%**)**	**Cases** **(n = 186)** **No. (**%**)**	***p* value** ^**a**^
**Demographic Characteristics**			
Age in years, mean (±SD)	60.9 (10.6)	58.9 (11.5)	0.096^∗^
Male	98 (57.3)	101 (55.2)	0.560
Female	73 (42.7)	85 (45.7)	-
**Systemic Diseases**			
Diabetes mellitus	65 (38.0)	75 (40.3)	0.654
Coronary artery disease	4 (2.3)	6 (3.2)	0.612
Hypertension	56 (32.7)	71 (38.1)	0.285
Hypercholesterolemia	8 (4.6)	14 (7.5)	0.263
**Health Awareness / Behavior**			
Family history of glaucoma	7 (4.1)	18 (9.6)	0.039
Smoking	15 (8.7)	20 (10.7)	0.527

^a^Pearson Chi^2^ test, ^*∗*^*t*-test.

**Table 2:** Association analysis of allele frequency and genotype distribution for SNP rs7916697 in POAG patients and controls.

**SNP (Gene)**	**rs7916697 (*ATOH7*)**				
	**Controls** **(n = 171)** **No. (**%**)**	**POAG** **(n = 186)** **No. (**%**)**	**Odds ratio**	**95**%** confidence interval**	***p* value** ^**a**^
**Allelic analysis**					
G	219 (64.0)	227 (61.0)	1	Reference	-
A^∗^	123 (36.0)	145 (39.0)	0.88	0.65 – 1.19	0.406
HWE P	0.968	0.936	-	-	-
**Genotype and Model analysis**					
G/G	70 (40.9)	69 (37.1)	1	Reference	-
G/A	79 (46.2)	89 (47.8)	0.87	0.55 – 1.37	0.560
A/A	22 (12.8)	28 (15.0)	0.77	0.40 – 1.48	0.438
Additive	-	-	-	-	0.707^§^
Dominant	-	-	0.85	0.55 – 1.30	0.458
Recessive	-	-	0.83	0.45 – 1.52	0.554

^a^Pearson Chi^2^ test. ^∗^Risk variant. HWE P, Hardy-Weinberg equilibrium *p* value. ^§^Fisher exact test.

**Table 3:** Analysis of genotype effect on demographic and clinical characteristics within PAOG group.

**Characteristics**	**Genotypes**			***p* value** ^**a**^
	**G/G** **(n= 69)** **No. (**%**)**	**G/A** **(n= 89)** **No. (**%**)**	**A/A** **(n= 28)** **No. (**%**)**	
**DEMOGRAPHIC**				
Age in years, mean (SD)	62.2 (9.7)	60.0 (11.0)	60.4 (11.4)	0.413^∗^
Male	35 (50.7)	50 (56.1)	16 (57.1)	0.750
Female	34 (49.3)	39 (43.8)	12 (42.8)	-
**MEDICAL HISTORY**				
Family history of glaucoma	6 (8.7)	7 (7.8)	5 (17.8)	0.278
Smoking	5 (7.2)	12 (13.5)	3 (10.7)	0.455
Diabetes mellitus	22 (31.9)	38 (42.7)	15 (53.5)	0.116
Hypertension	24 (34.8)	33(37.0)	14 (50.0)	0.360
Coronary artery disease	1 (1.4)	4 (4.5)	1 (3.5)	0.557
Hypercholesterolemia	3 (4.3)	7 (7.8)	4 (14.3)	0.240
**GLAUCOMA INDICES**				
Intraocular pressure in mmHg, mean (SD)	22.5 (8.9)	24.0 (9.7)	22.6 (7.9)	0.444^∗∗^
Cup/disc ratio	0.84 (0.6)	0.75 (0.2)	0.82 (0.1)	0.259^∗∗^
Number of anti-glaucoma medications	1.7 (1.1)	2.0 (1.0)	1.9 (1.1)	0.325^∗∗^

^a^Pearson Chi^2^ test. ^∗^One-way ANOVA. ^∗∗^Kruskal-Wallis test.

**Table 4:** Binary logistic regression analysis to assess the effect of age, sex, and genotype on disease outcome.

**Variables**	**Odds ratio**	**95**%** confidence interval**	***p* value**
Age	1.01	0.99 – 1.03	0.096
Sex^a^	0.91	0.59 – 1.39	0.676
Genotype^b^	-	-	0.627
G/A	1.17	0.74 – 1.83	0.499
A/A	1.34	0.70 – 2.59	0.371

^a^Female as reference, ^b^G/G as reference.

## Data Availability

The data supporting the conclusions of this article are all presented within the article.
